# *N*-(1,10-Phenanthrolin-5-yl)-4-(2-pyridyl)­benzamide monohydrate

**DOI:** 10.1107/S1600536808029851

**Published:** 2008-09-20

**Authors:** Masayuki Kobayashi, Shigeyuki Masaoka, Ken Sakai

**Affiliations:** a Department of Chemistry, Faculty of Science, Kyushu University, Hakozaki 6-10-1, Higashi-ku, Fukuoka 812-8581, Japan

## Abstract

In the title mol­ecule, C_24_H_16_N_4_O·H_2_O, the benzene ring of the 1,10-phenanthroline group and that of the 2-phenyl­pyridine group are respectively twisted by 67.9 (1) and 15.3 (3)° from the carbamoyl group defined by the plane of the O=C—N group of atoms. The water mol­ecule is hydrogen bonded to one of the phenanthroline N atoms. In the crystal structure, significant π–π stacking inter­actions occur, with centroid-to-centroid separations in the range 3.567–3.681 (2) Å.

## Related literature

For background information, see: Ozawa & Sakai (2007 ▶); Ozawa *et al.* (2006 ▶, 2007 ▶); Sakai & Ozawa (2007 ▶).
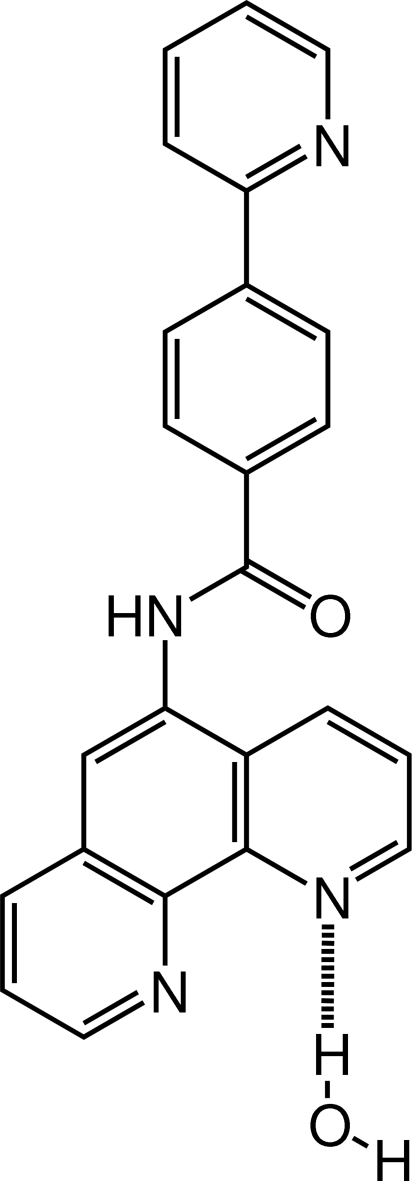


## Experimental

### 

#### Crystal data

C_24_H_16_N_4_O·H_2_O
*M* *_r_* = 394.42
Triclinic, 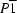

*a* = 8.226 (2) Å
*b* = 9.357 (3) Å
*c* = 13.849 (4) Å
α = 73.638 (3)°
β = 82.883 (4)°
γ = 64.695 (3)°
*V* = 924.7 (5) Å^3^
*Z* = 2
Mo *K*α radiation
μ = 0.09 mm^−1^
*T* = 296 (2) K
0.13 × 0.05 × 0.05 mm

#### Data collection

Bruker SMART APEX CCD-detector diffractometer
Absorption correction: multi-scan (*SADABS*; Sheldrick, 1996 ▶) *T*_min_ = 0.992, *T*_max_ = 0.995
8821 measured reflections
3222 independent reflections
2284 reflections with *I* > 2σ(*I*)
*R*_int_ = 0.036

#### Refinement

*R*[*F* ^2^ > 2σ(*F* ^2^)] = 0.047
*wR*(*F* ^2^) = 0.114
*S* = 1.03
3222 reflections
279 parameters
H atoms treated by a mixture of independent and constrained refinement
Δρ_max_ = 0.30 e Å^−3^
Δρ_min_ = −0.23 e Å^−3^

### 

Data collection: *APEX2* (Bruker, 2006 ▶); cell refinement: *SAINT* (Bruker, 2004 ▶); data reduction: *SAINT*; program(s) used to solve structure: *SHELXS97* (Sheldrick, 2008 ▶); program(s) used to refine structure: *SHELXL97* (Sheldrick, 2008 ▶); molecular graphics: *KENX* (Sakai, 2004 ▶); software used to prepare material for publication: *SHELXL97*, *TEXSAN* (Molecular Structure Corporation, 2001 ▶), *KENX* and *ORTEPII* (Johnson, 1976 ▶).

## Supplementary Material

Crystal structure: contains datablocks global, I. DOI: 10.1107/S1600536808029851/lh2685sup1.cif

Structure factors: contains datablocks I. DOI: 10.1107/S1600536808029851/lh2685Isup2.hkl

Additional supplementary materials:  crystallographic information; 3D view; checkCIF report

## Figures and Tables

**Table 1:** Hydrogen-bond geometry (Å, °)

*D*—H⋯*A*	*D*—H	H⋯*A*	*D*⋯*A*	*D*—H⋯*A*
O2—H1*S*⋯N2	0.92 (4)	2.01 (4)	2.905 (2)	163 (3)

**Fig. 1.**
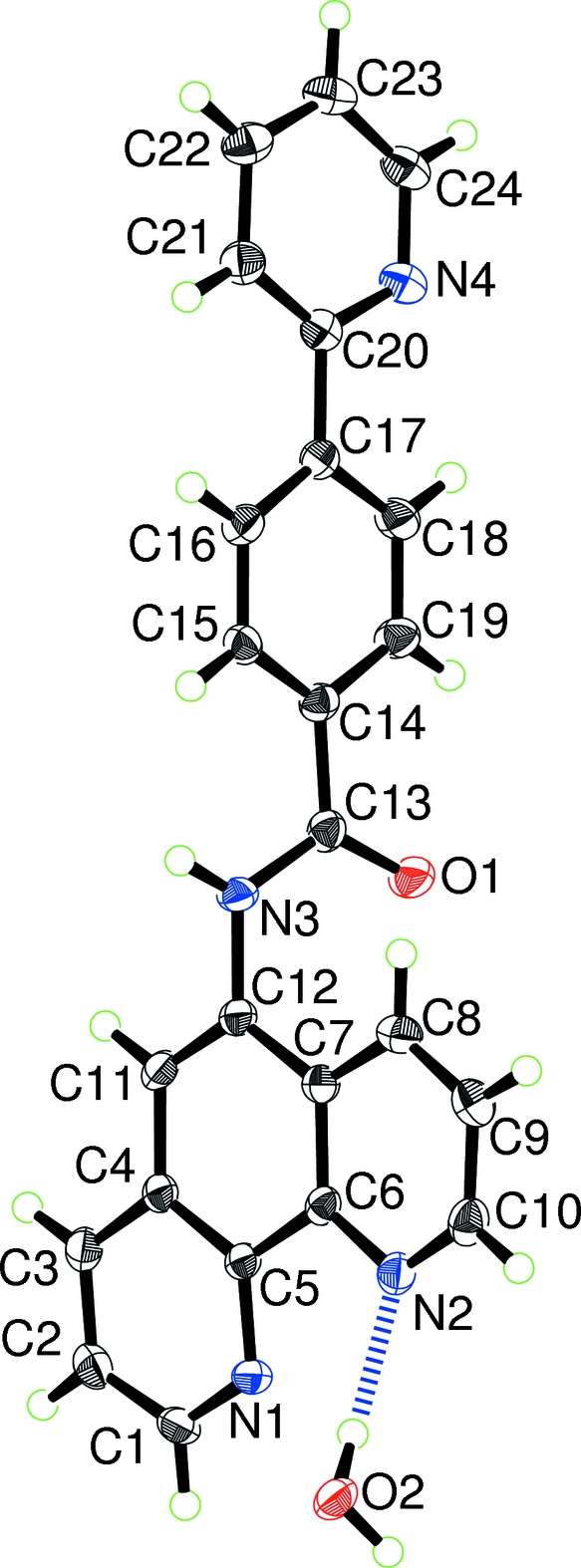
The molecular structure of (I) showing the atom-labeling scheme. Displacement ellipsoids are drawn at the 50% probability level.

**Fig. 2.**
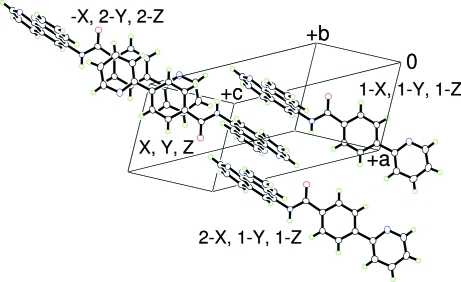
A view showing the manner how the aromatic moieties are stacked in the crystal. Water molecules are omitted for clarity.

**Fig. 3.**
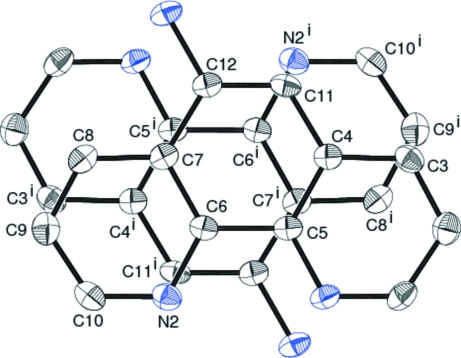
View showing the manner in whch the phen moieties of two symmetry related molecules are stacked, showing a view perpendicular to the planes stacked to each other. Only the labeled atoms are involved in the mean-plane calculations carried out to determine the interplanar separation between the two planes stacked with two different interactions within a one dimensional column. H atoms have been omitted for clarity. [Symmetry codes: (i) 1 - x, 1 - y, 1 - z; (ii) 2 - x, 1 - y, 1 - z].

**Fig. 4.**
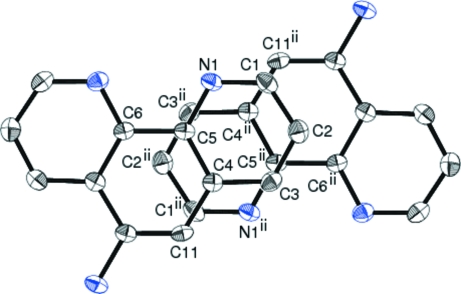
View showing the manner in whch the phen moieties of two symmetry related molecules are stacked, showing a view perpendicular to the planes stacked to each other. Only the labeled atoms are involved in the mean-plane calculations carried out to determine the interplanar separation between the two planes stacked with two different interactions within a one dimensional column. H atoms have been omitted for clarity. [Symmetry codes: (i) 1 - x, 1 - y, 1 - z; (ii) 2 - x, 1 - y, 1 - z].

## supplementary crystallographic information

### Comment

Interest has been focused on the development of photo-hydrogen-evolving molecular devices, which not only serve as a photosensitizing molecule but also as an H_2_-evolving catalyst (Ozawa *et al.*, 2006, 2007; Ozawa & Sakai, 2007; Sakai & Ozawa, 2007). One of the most important findings in these studies is that the visible light-induced reduction of water by edta (a sacrificial electron donor) into molecular hydrogen can be driven by a condensation product of [Ru(bpy)_2_(5-amino-phen)]^2+^ and PtCl_2_(4,4'-dicarboxy-bpy) (bpy = 2,2'-bipyridine; phen = 1,10-phenanthroline) with a quantum efficiency of *ca* 0.01. *N*-(1,10-phenanthrolin-5-yl)-4-carbamoyl-4'-carboxy-2,2'-bipyridine, which is considered a structural analog of the title compound (I), is employed as a bridging spacer connecting the two different metal centers (Ozawa *et al.*, 2006). In order to improve the quantum efficiency in the light-driven H_2_ formation, efforts have been made to clarify the mechanism of this photoinduced process and also to develop the more highly efficient photo-hydrogen-evolving molecular devices. The new bridging spacer (I) was prepared to evaluate the change in photocatalytic efficiency upon replacing the bpy attached to the Pt^II^ center with a phenylpyridinate ligand. We have already succeeded in preparing and testing the corresponding Ru^II^/Pt^II^ complex but, unfortunately, the compound was found to be ineffective towards the edta-based reduction of H_2_O into H_2_, which will be separately reported elsewhere in a future publication.

The molecular structure of (I) is shown in Fig. 1. The water is hydrogen bonded to one of the nitrogen atoms of the phen moiety (Table 1). The phen moiety is slightly deformed from an ideal planar geometry, presumably due to the π–π stacking interactions formed in the crystal, as discussed below. The C1–C3 and the C8–C10 groups of atoms are shifted from the central benzene plane of phen, defined with atoms C4–C7, C11 and C12, in such a manner that the C1–C3 and the C8–C10 units are shifted to opposite sides of the benzene plane. The two pyridyl planes within phen, *i.e.*, the planes defined with atoms N1 and C1–C5 and atoms N2 and C6–C10, are declined with respect to the central benzene plane by 3.7 (1) and 2.5 (1)°, respectively. The carbamoyl group defined by the plane of atoms C13, O1, and N3 is twisted with regard to the benzene ring of phen at an angle of 67.9 (1)°. The carbamoyl plane is also declined by 15.3 (3)° with respect to the benzene plane of the phenylpyridine moiety defined with atoms C14–C19. The dihedral angle between the plane defined with atoms C14–C19 and that with atoms N4 and C20–C24 is 5.6 (2)°, which corresponds to the dihedral angle of the two aromatic rings within the phenylpyridine moiety. In the best plane calculations carried out for the above-mentioned five aromatic rings, the 6-atom r.m.s. deviations are in the range of 0.003–0.015 Å, revealing that all these rings have an essentially planar geometry.

As shown in Fig. 2, the phen moiety has a π-stack to the adjacent phen moieties to give a one-dimensional stack in the crystal. As shown in Fig. 3, one is considered as a strong stack with almost full overlap of the phen moieties, while the other as a relatively weak stack based on the partial overlap of the phen moieties. The interplanar separations between the two aromatic systems for the former and the latter geometries are 3.52 (2) and 3.43 (2) Å, respectively. On the other hand, the phenylpyridine moiety forms a π-stack dimer with the interplanar separation 3.48 (11) Å.

### Experimental

A suspension of 4-(2-pyridyl)benzoic acid^.^0.5H_2_O (0.25 g, 1.2 mmol) in 10 ml of thionyl chloride was refluxed for 3 h. The resulting solution was evaporated to dryness and the residue was dried *in vacuo* to give 4-(2-pyridyl)benzoil chloride. This was dissolved in 20 ml of anhydrous CH_2_Cl_2_. To a solution of 5-amino-1,10-phenanthroline (0.20 g, 1.0 mmol) and triethylamine (0.5 ml) in a 1:1 mixture of anhydrous CH_2_Cl_2_ and anhydrous acetonitrile (100 ml) under cooling in an ice bath was added the former solution under Ar over 30 min. After stirring for 3 days at room temperature, the solution was evaporated to dryness. The residue was washed with aqueous 5% NaHCO_3_ solution (20 ml), and collected by filtration. The crude product was recrystallized from ethanol. Yield: 0.29 g (72%). Analysis calculated for C_24_H_16_N_4_O^.^1.5H_2_O: C, 71.45; H, 4.75; N; 13.89. Found: C, 71.30; H, 4.52; N, 13.90. ^1^H NMR (300.53 MHz, dmso-d_6_), p.p.m.: δ 10.75 (s, 1H), 9.14 (d, 1H, J = 4.24 Hz), 9.08 (d, 1H, J = 4.24 Hz), 8.73 (d, 1H, J = 4.96 Hz), 8.53 (t, 2H, J = 9.11 Hz), 8.32–8.23 (m, 4H), 8.15 (s, 1H), 8.12 (d, 1H, J = 6.97 Hz), 7.98–7.92 (m, 1H), 7.83–7.76 (m, 2H), 7.45–7.41 (m, 1H). ESI-TOF MS (positive ion, methanol): m/*z* 376.96 [*M* - 1.5H_2_O + H^+^]^+^. A good quality single-crystal was prepared by slow evaporation of a *N*,*N*-dimethylformamide (DMF) solution as follows. Compound (I) was dissolved in a minimum amount of DMF and the solution was left for several days at room temperature, during which the solution gradually reduced its volume to give crystals suitable for X-ray diffraction analysis.

### Refinement

Hydrogen atoms, except for those of a water molecule, were placed in their idealized positions (aromatic C—H = 0.93 Å and N—H = 0.86 Å), and included in the refinement in a riding-model approximation with *U*_iso_(H) = 1.2Ueq(C and N). Hydrogen atoms of a water molecule were refined isotropically. In the final difference Fourier map, the highest peak was located 0.63 Å from atom H3. The deepest hole was located 0.38 Å from atom H3.

### Crystal data

**Table:**

C_24_H_16_N_4_O·H_2_O	*Z* = 2
*M**_r_* = 394.42	*F*(000) = 412
Triclinic, *P*1	? # Insert any comments here.
Hall symbol: -P 1	*D*_x_ = 1.417 Mg m^−^^3^
*a* = 8.226 (2) Å	Mo *K*α radiation, λ = 0.71073 Å
*b* = 9.357 (3) Å	Cell parameters from 7706 reflections
*c* = 13.849 (4) Å	θ = 2.5–25.0°
α = 73.638 (3)°	µ = 0.09 mm^−^^1^
β = 82.883 (4)°	*T* = 296 K
γ = 64.695 (3)°	Cube, yellow
*V* = 924.7 (5) Å^3^	0.13 × 0.05 × 0.05 mm

### Data collection

**Table:**

Bruker SMART APEX CCD-detector diffractometer	3222 independent reflections
Radiation source: rotating anode with a mirror focusing unit	2284 reflections with *I* > 2σ(*I*)
graphite	*R*_int_ = 0.037
φ and ω scans	θ_max_ = 25.0°, θ_min_ = 2.5°
Absorption correction: multi-scan (*SADABS*; Sheldrick, 1996)	*h* = −9→9
*T*_min_ = 0.992, *T*_max_ = 0.995	*k* = −11→11
8821 measured reflections	*l* = −16→16

### Refinement

**Table:**

Refinement on *F*^2^	Primary atom site location: structure-invariant direct methods
Least-squares matrix: full	Secondary atom site location: difference Fourier map
*R*[*F*^2^ > 2σ(*F*^2^)] = 0.047	Hydrogen site location: inferred from neighbouring sites
*wR*(*F*^2^) = 0.114	H atoms treated by a mixture of independent and constrained refinement
*S* = 1.03	*w* = 1/[σ^2^(*F*_o_^2^) + (0.0447*P*)^2^ + 0.4023*P*] where *P* = (*F*_o_^2^ + 2*F*_c_^2^)/3
3222 reflections	(Δ/σ)_max_ < 0.001
279 parameters	Δρ_max_ = 0.30 e Å^−^^3^
0 restraints	Δρ_min_ = −0.23 e Å^−^^3^

### Special details

**Table:**

Experimental. The first 50 frames were rescanned at the end of data collection to evaluate any possible decay phenomenon. Since it was judged to be negligible, no decay correction was applied to the data.
Geometry. All e.s.d.'s (except the e.s.d. in the dihedral angle between two l.s. planes) are estimated using the full covariance matrix. The cell e.s.d.'s are taken into account individually in the estimation of e.s.d.'s in distances, angles and torsion angles; correlations between e.s.d.'s in cell parameters are only used when they are defined by crystal symmetry. An approximate (isotropic) treatment of cell e.s.d.'s is used for estimating e.s.d.'s involving l.s. planes.
Refinement. Refinement of *F*^2^ against ALL reflections. The weighted *R*-factor *wR* and goodness of fit *S* are based on *F*^2^, conventional *R*-factors *R* are based on *F*, with *F* set to zero for negative *F*^2^. The threshold expression of *F*^2^ > σ(*F*^2^) is used only for calculating *R*-factors(gt) *etc*. and is not relevant to the choice of reflections for refinement. *R*-factors based on *F*^2^ are statistically about twice as large as those based on *F*, and *R*- factors based on ALL data will be even larger.

### Fractional atomic coordinates and isotropic or equivalent isotropic displacement parameters (Å^2^)

**Table:**

	*x*	*y*	*z*	*U*_iso_*/*U*_eq_	
O1	0.6321 (2)	0.64738 (18)	0.79108 (12)	0.0325 (4)	
O2	0.8041 (2)	0.0010 (2)	0.48055 (13)	0.0320 (4)	
N1	0.9204 (2)	0.3157 (2)	0.44978 (14)	0.0227 (4)	
N2	0.7555 (2)	0.2000 (2)	0.61822 (14)	0.0240 (4)	
N3	0.4357 (2)	0.7537 (2)	0.66380 (13)	0.0234 (4)	
H3	0.3311	0.8231	0.6404	0.028*	
N4	0.1196 (2)	1.2126 (2)	1.05472 (14)	0.0283 (5)	
C1	0.9942 (3)	0.3743 (3)	0.36656 (17)	0.0261 (5)	
H1	1.0806	0.3002	0.3340	0.031*	
C2	0.9506 (3)	0.5398 (3)	0.32478 (17)	0.0269 (5)	
H2	1.0048	0.5749	0.2654	0.032*	
C3	0.8266 (3)	0.6497 (3)	0.37276 (17)	0.0242 (5)	
H3A	0.7957	0.7610	0.3466	0.029*	
C4	0.7459 (3)	0.5937 (2)	0.46189 (16)	0.0202 (5)	
C5	0.7961 (3)	0.4238 (2)	0.49721 (16)	0.0194 (5)	
C6	0.7149 (3)	0.3620 (2)	0.58924 (16)	0.0196 (5)	
C7	0.5959 (3)	0.4720 (2)	0.64404 (16)	0.0203 (5)	
C8	0.5206 (3)	0.4072 (3)	0.73217 (17)	0.0251 (5)	
H8	0.4421	0.4754	0.7708	0.030*	
C9	0.5626 (3)	0.2438 (3)	0.76114 (18)	0.0281 (5)	
H9	0.5137	0.1991	0.8197	0.034*	
C10	0.6796 (3)	0.1450 (3)	0.70180 (18)	0.0274 (5)	
H10	0.7061	0.0338	0.7219	0.033*	
C11	0.6237 (3)	0.7014 (2)	0.51907 (17)	0.0222 (5)	
H11	0.5914	0.8133	0.4952	0.027*	
C12	0.5542 (3)	0.6440 (2)	0.60716 (16)	0.0207 (5)	
C13	0.4863 (3)	0.7494 (3)	0.75393 (17)	0.0235 (5)	
C14	0.3587 (3)	0.8709 (2)	0.80803 (16)	0.0214 (5)	
C15	0.1777 (3)	0.9621 (2)	0.78548 (17)	0.0226 (5)	
H15	0.1289	0.9500	0.7328	0.027*	
C16	0.0692 (3)	1.0709 (3)	0.84065 (17)	0.0241 (5)	
H16	−0.0519	1.1309	0.8245	0.029*	
C17	0.1378 (3)	1.0925 (2)	0.92009 (16)	0.0214 (5)	
C18	0.3183 (3)	0.9973 (3)	0.94320 (18)	0.0273 (5)	
H18	0.3666	1.0069	0.9971	0.033*	
C19	0.4274 (3)	0.8894 (3)	0.88841 (17)	0.0267 (5)	
H19	0.5482	0.8282	0.9052	0.032*	
C20	0.0297 (3)	1.2124 (3)	0.97979 (17)	0.0222 (5)	
C21	−0.1468 (3)	1.3208 (3)	0.95877 (18)	0.0271 (5)	
H21	−0.2065	1.3184	0.9069	0.033*	
C22	−0.2342 (3)	1.4330 (3)	1.01517 (18)	0.0303 (6)	
H22	−0.3535	1.5064	1.0021	0.036*	
C23	−0.1423 (3)	1.4343 (3)	1.09085 (18)	0.0297 (6)	
H23	−0.1972	1.5092	1.1297	0.036*	
C24	0.0327 (3)	1.3224 (3)	1.10773 (18)	0.0303 (6)	
H24	0.0944	1.3232	1.1593	0.036*	
H1S	0.809 (4)	0.067 (4)	0.518 (3)	0.093 (12)*	
H2S	0.900 (4)	−0.092 (4)	0.494 (2)	0.068 (10)*	

### Atomic displacement parameters (Å^2^)

**Table:**

	*U*^11^	*U*^22^	*U*^33^	*U*^12^	*U*^13^	*U*^23^
O1	0.0266 (9)	0.0314 (9)	0.0339 (10)	−0.0004 (8)	−0.0089 (8)	−0.0150 (8)
O2	0.0348 (10)	0.0202 (9)	0.0357 (11)	−0.0016 (8)	−0.0115 (8)	−0.0102 (8)
N1	0.0229 (10)	0.0218 (10)	0.0218 (11)	−0.0049 (8)	−0.0011 (8)	−0.0096 (8)
N2	0.0272 (10)	0.0207 (10)	0.0235 (11)	−0.0087 (8)	−0.0050 (9)	−0.0045 (8)
N3	0.0183 (10)	0.0242 (10)	0.0249 (11)	−0.0022 (8)	−0.0025 (8)	−0.0116 (8)
N4	0.0317 (11)	0.0307 (11)	0.0263 (12)	−0.0140 (9)	0.0018 (9)	−0.0120 (9)
C1	0.0248 (13)	0.0314 (13)	0.0209 (14)	−0.0075 (10)	0.0008 (10)	−0.0119 (11)
C2	0.0279 (13)	0.0325 (13)	0.0200 (13)	−0.0125 (11)	−0.0026 (10)	−0.0053 (11)
C3	0.0281 (13)	0.0208 (11)	0.0229 (14)	−0.0099 (10)	−0.0055 (10)	−0.0023 (10)
C4	0.0189 (11)	0.0209 (11)	0.0202 (13)	−0.0064 (9)	−0.0050 (9)	−0.0053 (9)
C5	0.0180 (11)	0.0190 (11)	0.0209 (13)	−0.0050 (9)	−0.0048 (9)	−0.0067 (10)
C6	0.0187 (11)	0.0182 (11)	0.0217 (13)	−0.0063 (9)	−0.0051 (9)	−0.0047 (9)
C7	0.0164 (11)	0.0234 (11)	0.0212 (13)	−0.0064 (9)	−0.0038 (9)	−0.0073 (10)
C8	0.0225 (12)	0.0290 (12)	0.0258 (14)	−0.0102 (10)	0.0024 (10)	−0.0118 (10)
C9	0.0304 (13)	0.0328 (13)	0.0237 (14)	−0.0166 (11)	0.0003 (11)	−0.0055 (11)
C10	0.0322 (13)	0.0213 (11)	0.0286 (15)	−0.0124 (10)	−0.0048 (11)	−0.0020 (11)
C11	0.0218 (12)	0.0152 (11)	0.0265 (14)	−0.0039 (9)	−0.0060 (10)	−0.0044 (10)
C12	0.0177 (11)	0.0211 (11)	0.0222 (13)	−0.0047 (9)	−0.0041 (10)	−0.0075 (10)
C13	0.0222 (12)	0.0225 (12)	0.0250 (14)	−0.0074 (10)	−0.0051 (10)	−0.0055 (10)
C14	0.0232 (12)	0.0179 (11)	0.0233 (13)	−0.0092 (9)	0.0014 (10)	−0.0050 (10)
C15	0.0267 (12)	0.0220 (11)	0.0202 (13)	−0.0104 (10)	−0.0020 (10)	−0.0057 (10)
C16	0.0215 (12)	0.0219 (11)	0.0285 (14)	−0.0074 (10)	−0.0022 (10)	−0.0073 (10)
C17	0.0248 (12)	0.0184 (11)	0.0224 (13)	−0.0109 (10)	0.0004 (10)	−0.0043 (10)
C18	0.0289 (13)	0.0304 (13)	0.0271 (14)	−0.0120 (11)	−0.0042 (11)	−0.0127 (11)
C19	0.0225 (12)	0.0265 (12)	0.0315 (15)	−0.0072 (10)	−0.0026 (10)	−0.0117 (11)
C20	0.0262 (12)	0.0219 (11)	0.0224 (13)	−0.0150 (10)	0.0027 (10)	−0.0046 (10)
C21	0.0278 (13)	0.0270 (12)	0.0281 (14)	−0.0121 (10)	−0.0003 (11)	−0.0081 (11)
C22	0.0257 (13)	0.0285 (13)	0.0364 (15)	−0.0103 (10)	0.0059 (11)	−0.0116 (11)
C23	0.0321 (14)	0.0325 (13)	0.0310 (15)	−0.0168 (11)	0.0106 (11)	−0.0166 (11)
C24	0.0373 (15)	0.0349 (14)	0.0257 (14)	−0.0188 (12)	0.0028 (11)	−0.0133 (11)

### Geometric parameters (Å, °)

**Table:**

O1—C13	1.229 (2)	C9—C10	1.388 (3)
O2—H1S	0.92 (4)	C9—H9	0.9300
O2—H2S	0.88 (3)	C10—H10	0.9300
N1—C1	1.324 (3)	C11—C12	1.351 (3)
N1—C5	1.351 (3)	C11—H11	0.9300
N2—C10	1.325 (3)	C13—C14	1.490 (3)
N2—C6	1.352 (3)	C14—C15	1.386 (3)
N3—C13	1.349 (3)	C14—C19	1.392 (3)
N3—C12	1.424 (3)	C15—C16	1.381 (3)
N3—H3	0.8600	C15—H15	0.9300
N4—C24	1.331 (3)	C16—C17	1.395 (3)
N4—C20	1.347 (3)	C16—H16	0.9300
C1—C2	1.391 (3)	C17—C18	1.388 (3)
C1—H1	0.9300	C17—C20	1.490 (3)
C2—C3	1.363 (3)	C18—C19	1.375 (3)
C2—H2	0.9300	C18—H18	0.9300
C3—C4	1.403 (3)	C19—H19	0.9300
C3—H3A	0.9300	C20—C21	1.378 (3)
C4—C5	1.410 (3)	C21—C22	1.381 (3)
C4—C11	1.428 (3)	C21—H21	0.9300
C5—C6	1.452 (3)	C22—C23	1.372 (3)
C6—C7	1.410 (3)	C22—H22	0.9300
C7—C8	1.400 (3)	C23—C24	1.371 (3)
C7—C12	1.441 (3)	C23—H23	0.9300
C8—C9	1.362 (3)	C24—H24	0.9300
C8—H8	0.9300		
			
H1S—O2—H2S	109 (3)	C11—C12—N3	120.22 (19)
C1—N1—C5	117.71 (19)	C11—C12—C7	120.60 (19)
C10—N2—C6	117.68 (19)	N3—C12—C7	119.2 (2)
C13—N3—C12	120.42 (17)	O1—C13—N3	121.2 (2)
C13—N3—H3	119.8	O1—C13—C14	120.86 (19)
C12—N3—H3	119.8	N3—C13—C14	117.90 (18)
C24—N4—C20	117.8 (2)	C15—C14—C19	118.5 (2)
N1—C1—C2	124.1 (2)	C15—C14—C13	124.69 (19)
N1—C1—H1	118.0	C19—C14—C13	116.80 (19)
C2—C1—H1	118.0	C16—C15—C14	120.5 (2)
C3—C2—C1	118.5 (2)	C16—C15—H15	119.7
C3—C2—H2	120.7	C14—C15—H15	119.7
C1—C2—H2	120.7	C15—C16—C17	121.3 (2)
C2—C3—C4	119.6 (2)	C15—C16—H16	119.3
C2—C3—H3A	120.2	C17—C16—H16	119.3
C4—C3—H3A	120.2	C18—C17—C16	117.4 (2)
C3—C4—C5	117.66 (19)	C18—C17—C20	118.70 (19)
C3—C4—C11	122.32 (19)	C16—C17—C20	123.92 (19)
C5—C4—C11	119.9 (2)	C19—C18—C17	121.7 (2)
N1—C5—C4	122.4 (2)	C19—C18—H18	119.2
N1—C5—C6	118.61 (18)	C17—C18—H18	119.2
C4—C5—C6	119.02 (19)	C18—C19—C14	120.5 (2)
N2—C6—C7	122.6 (2)	C18—C19—H19	119.7
N2—C6—C5	118.13 (19)	C14—C19—H19	119.7
C7—C6—C5	119.30 (19)	N4—C20—C21	121.5 (2)
C8—C7—C6	117.37 (19)	N4—C20—C17	114.92 (19)
C8—C7—C12	122.94 (19)	C21—C20—C17	123.5 (2)
C6—C7—C12	119.7 (2)	C20—C21—C22	119.6 (2)
C9—C8—C7	119.7 (2)	C20—C21—H21	120.2
C9—C8—H8	120.1	C22—C21—H21	120.2
C7—C8—H8	120.1	C23—C22—C21	118.9 (2)
C8—C9—C10	118.9 (2)	C23—C22—H22	120.6
C8—C9—H9	120.6	C21—C22—H22	120.6
C10—C9—H9	120.6	C24—C23—C22	118.2 (2)
N2—C10—C9	123.8 (2)	C24—C23—H23	120.9
N2—C10—H10	118.1	C22—C23—H23	120.9
C9—C10—H10	118.1	N4—C24—C23	124.0 (2)
C12—C11—C4	121.33 (19)	N4—C24—H24	118.0
C12—C11—H11	119.3	C23—C24—H24	118.0
C4—C11—H11	119.3		
			
C5—N1—C1—C2	−0.5 (3)	C8—C7—C12—C11	−176.69 (19)
N1—C1—C2—C3	1.2 (3)	C6—C7—C12—C11	2.1 (3)
C1—C2—C3—C4	−0.4 (3)	C8—C7—C12—N3	2.0 (3)
C2—C3—C4—C5	−0.9 (3)	C6—C7—C12—N3	−179.23 (17)
C2—C3—C4—C11	175.97 (19)	C12—N3—C13—O1	−2.9 (3)
C1—N1—C5—C4	−0.9 (3)	C12—N3—C13—C14	178.02 (19)
C1—N1—C5—C6	−179.31 (17)	O1—C13—C14—C15	−163.3 (2)
C3—C4—C5—N1	1.6 (3)	N3—C13—C14—C15	15.8 (3)
C11—C4—C5—N1	−175.33 (18)	O1—C13—C14—C19	15.0 (3)
C3—C4—C5—C6	−179.97 (17)	N3—C13—C14—C19	−165.89 (19)
C11—C4—C5—C6	3.1 (3)	C19—C14—C15—C16	1.1 (3)
C10—N2—C6—C7	−0.3 (3)	C13—C14—C15—C16	179.4 (2)
C10—N2—C6—C5	−179.43 (18)	C14—C15—C16—C17	0.1 (3)
N1—C5—C6—N2	−6.0 (3)	C15—C16—C17—C18	−1.6 (3)
C4—C5—C6—N2	175.59 (17)	C15—C16—C17—C20	177.5 (2)
N1—C5—C6—C7	174.89 (18)	C16—C17—C18—C19	2.0 (3)
C4—C5—C6—C7	−3.6 (3)	C20—C17—C18—C19	−177.2 (2)
N2—C6—C7—C8	0.8 (3)	C17—C18—C19—C14	−0.8 (4)
C5—C6—C7—C8	179.88 (17)	C15—C14—C19—C18	−0.8 (3)
N2—C6—C7—C12	−178.05 (18)	C13—C14—C19—C18	−179.2 (2)
C5—C6—C7—C12	1.1 (3)	C24—N4—C20—C21	−0.6 (3)
C6—C7—C8—C9	−0.5 (3)	C24—N4—C20—C17	176.9 (2)
C12—C7—C8—C9	178.31 (19)	C18—C17—C20—N4	−2.1 (3)
C7—C8—C9—C10	−0.2 (3)	C16—C17—C20—N4	178.8 (2)
C6—N2—C10—C9	−0.5 (3)	C18—C17—C20—C21	175.3 (2)
C8—C9—C10—N2	0.8 (3)	C16—C17—C20—C21	−3.8 (3)
C3—C4—C11—C12	−176.79 (19)	N4—C20—C21—C22	0.3 (3)
C5—C4—C11—C12	0.0 (3)	C17—C20—C21—C22	−177.0 (2)
C4—C11—C12—N3	178.66 (18)	C20—C21—C22—C23	0.4 (3)
C4—C11—C12—C7	−2.6 (3)	C21—C22—C23—C24	−0.7 (3)
C13—N3—C12—C11	−112.9 (2)	C20—N4—C24—C23	0.3 (3)
C13—N3—C12—C7	68.4 (3)	C22—C23—C24—N4	0.4 (4)

### Hydrogen-bond geometry (Å, °)

**Table:**

*D*—H···*A*	*D*—H	H···*A*	*D*···*A*	*D*—H···*A*
O2—H1S···N2	0.92 (4)	2.01 (4)	2.905 (2)	163 (3)
